# Linking renal hypoxia and oxidative stress in chronic kidney disease: Based on clinical subjects and animal models

**DOI:** 10.17305/bb.2024.10257

**Published:** 2024-10-01

**Authors:** Yizeng Xu, Fang Lu, Meng Wang, Lingchen Wang, Chaoyang Ye, Shuohui Yang, Chen Wang

**Affiliations:** 1 Department of Nephrology, Shuguang Hospital Affiliated to Shanghai University of Traditional Chinese Medicine, Shanghai, China; 2 Key Laboratory of Liver and Kidney Diseases, Ministry of Education, Shanghai University of Traditional Chinese Medicine, Shanghai, China; 3 TCM Institute of Kidney Disease, Shanghai University of Traditional Chinese Medicine, Shanghai, China; 4 Shanghai Key Laboratory of Traditional Chinese Clinical Medicine, Shanghai University of Traditional Chinese Medicine, Shanghai, China; 5 Department of Radiology, Shuguang Hospital Affiliated to Shanghai University of Traditional Chinese Medicine, Shanghai, China; 6 Department of Radiology, Shanghai Municipal Hospital of Traditional Chinese Medicine, Shanghai University of Traditional Chinese Medicine, Shanghai, China

**Keywords:** Renal hypoxia, oxidative stress, chronic kidney disease (CKD), blood oxygenation level-dependent magnetic resonance imaging (BOLD-MRI).

## Abstract

The prevalence of chronic hypoxia and oxidative stress plays a key role in the progression of chronic kidney disease (CKD), but the underlying correlations between them need further elucidation. This study aims to explore the relationships between renal function, hypoxia, and oxidative stress in CKD. Seventy-six non-dialysis patients with CKD stages 1–5 and 8 healthy subjects were included in the clinical research. They were divided into 3 groups: healthy subjects, CKD stages 1–3, and CKD stages 4–5. In the animal study, 16 rat models of CKD were established through 5/6 renal ablation/infarction (A/I) surgery, and 8 normal rats were split into 3 groups: Sham, CKD, and losartan groups. Blood oxygenation level-dependent magnetic resonance imaging (BOLD-MRI) was used to measure cortical and medullary T2^*^ values (COT2^*^ and MET2^*^) in all subjects and rats to evaluate renal oxygenation. Biochemical indicators were used to assess renal function and antioxidant capacity. Furthermore, the effects of losartan on renal fibrosis, hypoxia, and oxidative stress were examined using immunoblotting, colorimetric, and fluorometric assays. The results demonstrated significant positive associations between COT2* and MET2* with estimated glomerular filtration rate (eGFR). Patients with CKD stages 4–5 showed significantly lower serum superoxide dismutase (SOD) levels, which also had positive correlations with eGFR, COT2*, and MET2*. Furthermore, losartan treatment resulted in improved renal function and fibrosis, leading to increased levels of COT2*, MET2*, and SOD levels in 5/6 A/I rats. This was accompanied by reduced levels of hypoxia-inducible factor-1 alpha (HIF-1α) and malondialdehyde (MDA). Furthermore, losartan restored the expression of nuclear factor erythroid 2-related factor 2 (Nrf2) and heme oxygenase-1 (HO-1), and suppressed the expression of Kelch-like ECH-associated protein 1 (Keap1) in 5/6 A/I kidneys. The study indicates that a decline in renal oxygenation and antioxidant capacity is associated with the severity of renal failure in CKD. Losartan can potentially alleviate renal hypoxia and oxidative stress in the treatment of CKD via the Keap1-Nrf2/HO-1 pathway.

## Introduction

Chronic kidney disease (CKD) remains a global health problem with significant morbidity and mortality [1]. Renal fibrosis is a common pathological basis of CKD, regardless of the initial causative factors [2]. Accumulating evidence suggests that renal hypoxia plays an important role in the progression of CKD and renal fibrosis [3]. Owing to the unique borderline hypoxia of the kidney in a physiological state, it is sensitive to oxygenation dyshomeostasis. In addition, various pathological conditions across CKD progression, such as oxidative stress, inflammation, and dysregulated angiogenesis, aggravate renal hypoxia [4, 5]. Several studies have found that renal hypoxia was present in different rat models of CKD, such as unilateral ureteral obstruction (UUO), 5/6 nephrectomy, and adenine-induced models [6–8]. Thus, renal hypoxia may be an independent predictor of renal injury in patients with CKD.

Oxidative stress, defined as an imbalance between oxidants and antioxidants, is prevalent during CKD progression and promotes renal injury. The kidney is a highly metabolic organ with abundant mitochondria, which makes it vulnerable to damage from oxidative stress [9]. It is difficult to separate oxidative stress and hypoxia in CKD because oxidative stress increases renal oxygen consumption. In turn, chronic hypoxia induces overproduction of reactive oxygen species (ROS), which aggravates oxidative stress [10]. Therefore, oxidative stress and hypoxia are marked features of CKD and major mediators of CKD progression.

**Figure 1.**
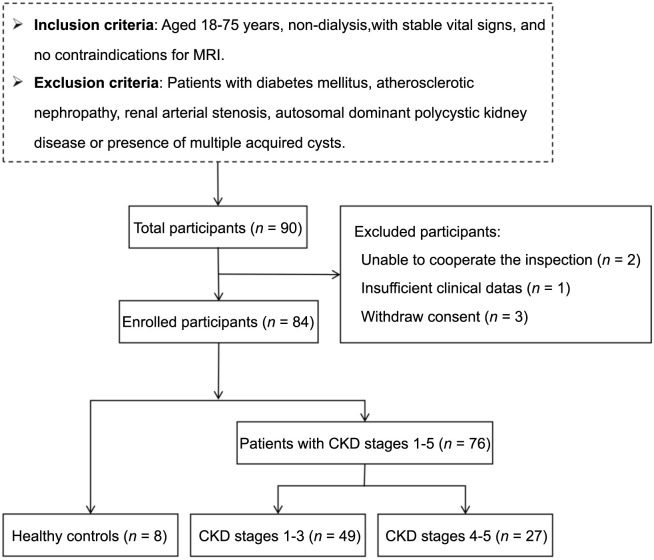
**Flowchart for the inclusion of subjects**. CKD: Chronic kidney disease; MRI: Magentic resonance imaging.

Blood oxygenation level-dependent magnetic resonance imaging (BOLD-MRI) offers a noninvasive and real-time method to evaluate renal oxygenation using MR parameters, including the effective transverse relaxation time T2^*^ and effective transverse relaxation rate R2^*^ (R2^*^ ═ 1/T2^*^) [11]. In previous clinical studies, we demonstrated the correlation between renal function and renal oxygenation in patients with CKD using BOLD-MRI [12, 13]. Next, we focused on the pathological factors that induce renal hypoxia in the progression of CKD. Hence, in the current study, we used both patients with CKD in the clinical trial and rodent models of CKD in the experiment to evaluate renal oxygenation by BOLD-MRI and detect antioxidant capacity and renal function through biochemical indices, in order to further explore the associations among hypoxia, oxidative stress, and renal function in CKD.

## Materials and methods

### Participant characteristics

CKD was diagnosed according to the National Kidney Foundation’s Kidney Disease Outcomes Quality Initiative (K/DOQI) [14]. The detailed inclusion and exclusion criteria are shown in (Figure 1).

A total of 76 adult patients with CKD and 8 healthy controls (HCs) were recruited between July 2020 and December 2021 at the Department of Nephrology in Shanghai Shuguang Hospital. All participants were asked to maintain a low dietary sodium intake within three days of MRI preparation. Biochemical indicators, including kidney function, serum superoxide dismutase (SOD), and hemoglobin, were measured prior to MRI scanning. The estimated glomerular filtration rate (eGFR) was calculated using the CKD Epidemiology Collaboration (CKD-EPI) equation.

### Animal and drug preparation

Male Wistar rats weighing 160–180 g were purchased from the SLAC Laboratory Animal Co., Ltd., (License No. SYXK 2022-0012) and raised in the experimental animal center of SHUTCM with a standard laboratory environment and diet. Losartan (100 mg/tablet) was purchased from Merck Sharp & Dohme Pharmaceutical Co., Ltd. (Hangzhou, China).

### Animal study protocol

**Figure 2.**
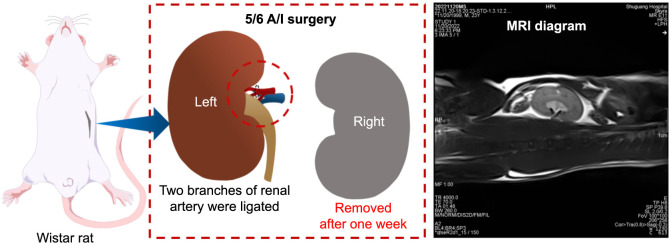
**Establishment of the 5/6 A/I rat model of CKD.** The MRI diagram was the T2^*^WI in the coronal plane of the 5/6 A/I kidney. A/I: Ablation/infarction; CKD: Chronic kidney disease; MRI: Magentic resonance imaging; T2*WI: Bold T2-weighted imaging.

Following a week of adaptive feeding, a rat model of CKD was established using the previously described 5/6 renal ablation/infarction (A/I) surgery [15]. Briefly, rats were anesthetized with pentobarbital sodium (40 mg/kg, i.p.) and placed on a thermostatic table. To expose the left kidney, a 1.0 cm long incision was made 0.5 cm below the left costal arch. The left renal artery and vein were carefully separated and two-thirds of the renal artery branches (posterior and anterior descending) were ligated. One week later, the right kidney was removed. The surgical procedure is depicted in Figure 2. Four weeks after surgery, 16 rat models of CKD underwent blood routine, and liver and kidney function tests, and were randomly assigned to either the CKD group (*n* ═ 8) or the CKD + losartan (LOS) group (*n* ═ 8). The remaining eight rats were divided into a sham-operated (Sham) group. The Sham rats received anesthesia and manipulated both renal pedicles, without damaging any renal parenchyma and vessels. The drug administration of losartan (20 mg/kg/day) was determined according to a previous study [15], and the Sham and CKD groups were treated with the same amount of distilled water as a control. The drugs were given by daily gavage for eight weeks.

At the end of the 8-week intervention, all rats underwent BOLD-MRI scanning, and 24-h total urine was collected using metabolic cages to detect 24-h urinary protein (24-h Upr) and the urinary albumin:creatinine ratio (UACR). Kidney function, serum SOD levels, and routine blood tests were performed at the same time. The creatinine clearance rate (Ccr), which is the eGFR, was calculated as follows: Ccr (mL/min) ═ urine creatinine (µmol/L) × 24-h urine volume (mL)/[serum creatinine (µmol/L) × 1440 (min)] [16]. Finally, all animals were anesthetized with pentobarbital sodium (40 mg/kg, i.p.), and remnant kidney tissues were acquired for histological and molecular studies.

### MRI acquisition and analysis

Before undergoing an MRI examination, the participants and rats fasted for 4–6 h. Rats were anesthetized by intraperitoneal injection of pentobarbital sodium and fixed with an animal-specific coil to maintain a prone position (Figure 4A). T1-weighted imaging (T1WI), T2-weighted imaging (T2WI), and T2^*^WI (BOLD) imaging of all kidneys were performed on a 3.0 T magnetic resonance scanner (MAGNETOM Skyra; Siemens Healthcare, Erlangen, Germany), as previously reported [12, 13]. A coronal multi-echo (7 echoes) gradient echo sequence was used for all subjects with echo times of 2.46, 4.92, 7.38, 9.84, 12.30, 14.76, and 17.22 ms; repetition time, 232 ms; slice thickness, 3.5 mm; flip angle, 60∘; bandwidth, 470 Hz/Px; field of view, 380 mm; and 168 × 256 matrix. Rats used a coronal multi-echo (5 echoes) gradient-echo sequence with echo times of 4.36, 11.90, 19.44, 29.68, and 34.52 ms; a repetition time of 417 ms; a voxel size of 0.2 mm × 0.2 mm × 2 mm; flip angle, 60∘; bandwidth, 260 Hz/Px; field of view, 120 mm; 205 × 256 matrix; and scanning time, 47 s.

**Figure 3.**
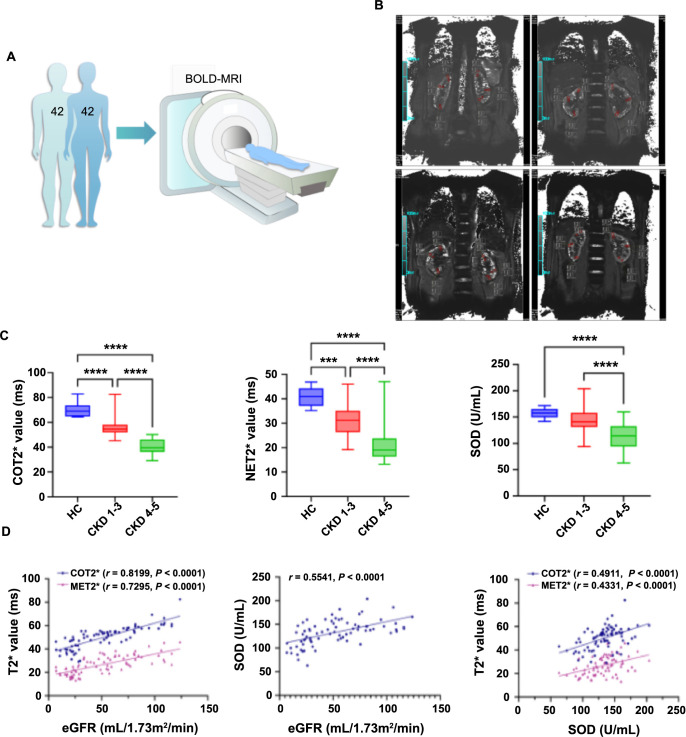
**Correlations of renal oxygenation and antioxidant capacity with renal function in patients with CKD.** (A) Schematic diagram of the evaluation of renal oxygenation by BOLD-MRI in participants; (B) BOLD-MRI T2^*^WI and T2^*^ map in the coronal plane of participants; (C) Comparisons of BOLD-MRI parameters and SOD among three groups (analyzed by one-way ANOVA); (D) Correlation of BOLD-MRI parameters, SOD, and eGFR (analyzed by Pearson’s correlation coefficient). Values for comparison are mean ± SD. ***P < 0.001, ****P < 0.0001. HC: Healthy controls; COT2^*^: Cortical T2^*^; MET2^*^: Medullary T2^*^; SOD: Serum superoxide dismutase; eGFR: Estimated glomerular filtration rate; CKD: Chronic kidney disease; BOLD-MRI: Blood oxygenation level-dependent magnetic resonance imaging; ANOVA: Analysis of variance.

**Figure 4.**
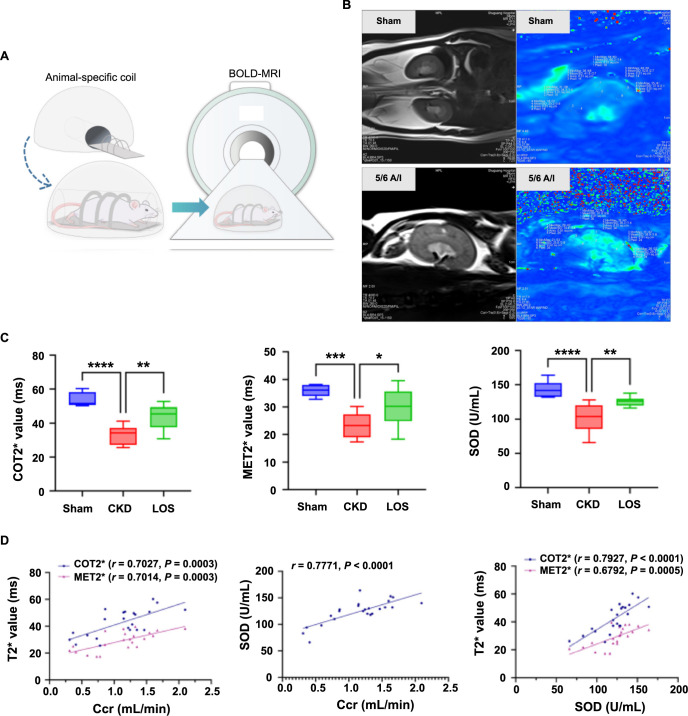
**Correlations of renal oxygenation and antioxidant capacity with renal function in CKD rats.** (A) Schematic diagram of the evaluation of renal oxygenation by BOLD-MRI in rats; (B) BOLD-MRI T2^*^WI and T2^*^ map in the coronal plane of the Sham and 5/6 A/I kidneys; (C) Comparisons of BOLD-MRI parameters and SOD among three groups (analyzed by one-way ANOVA); (D) Correlation of BOLD-MRI parameters, SOD, and Ccr (analyzed by Pearson’s correlation coefficient). Values for comparison are mean ± SD. ^*^P < 0.05, **P < 0.01, ***P < 0.001, ****P < 0.0001. LOS: Losartan; COT2^*^: Cortical T2^*^; MET2^*^: Medullary T2^*^; SOD: Serum superoxide dismutase; Ccr: Creatinine clearance rate; CKD: Chronic kidney disease; A/I: Ablation/infarction; BOLD-MRI: Blood oxygenation level-dependent magnetic resonance imaging; ANOVA: Analysis of variance.

After acquiring T2^*^WI maps of each kidney, T2^*^ values of renal cortex and medulla (COT2^*^ and MET2^*^) were evaluated by two experienced radiologists using six regions of interest (ROIs) with 0.01 cm^2^ placed at the upper, middle, and lower areas of the central slice, as shown in (Figures 3B and 4B). For participants, COT2^*^ and MET2^*^ were obtained from bilateral kidneys (average), and for rats, from the left kidneys. Both radiologists were blinded to the information on all participants and rats, including history, laboratory test results, staging of patients, and intervention of rats.

### Histopathological and immunofluorescence staining examination

The excised rat kidneys were fixed in 4% paraformaldehyde and embedded in paraffin. Paraffin-embedded sections (3-µm thickness) were subjected to hematoxylin–eosin (HE) and Masson’s trichrome staining according to the standard protocol, and observed under a microscope (Nikon Eclipse 80i, Japan) at 200× magnification. A semiquantitative scoring method was used to assess tubular injury in HE-stained sections, including tubular dilatation, atrophy, and detachment of tubular epithelial cells [17]: 0 represents no tubular injury; scores 1, 2, 3, and 4 represent injury involving < 25%, 25%–50%, 51%–75%, and > 75% of injured tubules, respectively. The severity of renal fibrosis was evaluated using ImageJ v.1.53 (National Institutes of Health, USA) according to the % fibrotic area in four randomly selected fields in each Masson’s trichrome section of the kidney.

For immunofluorescence staining, antigens were retrieved using the microwave EDTA buffer antigen retrieval method and blocked with 3% bovine serum albumin (BSA) and 0.1% Triton-100 after quenching autofluorescence. Sections (3-µm thick) were incubated with anti-hypoxia-inducible factor-1 alpha (HIF-1α) (1:200) overnight at 4 ^∘^C, followed by staining with FITC-labeled goat anti-rabbit IgG (Beyotime, China) as the secondary antibody. Positive staining was observed using fluorescence microscopy (Nikon Eclipse 80i, Japan) at 200× magnification.

### Western blot analysis

To extract proteins from the left kidney tissues, RIPA lysis buffer with protease and phosphatase inhibitors was used. The concentration of proteins was determined through the bicinchoninic acid (BCA) method. Protein samples were separated on 8% sodium dodecyl sulfate-polyacrylamide gels and electrotransferred onto polyvinylidene difluoride (PVDF) membranes. After incubation with blocking buffer for 1 h, the PVDF membranes were incubated with anti-FN (1:2000), anti-Col-I (1:2000), anti-α-SMA (1:2000), anti-HIF-1α (1:2000), anti-Mn-SOD (1:2000), anti-Kelch-like ECH-associated protein 1 (Keap1) (1:1000), anti-Nrf2 (1:1000), anti-heme oxygenase-1 (HO-1) (1:2000), and anti-GAPDH (1:3000) overnight at 4 ^∘^C. The signals were detected using the Luminescent Imaging Workstation (Tanon, China) and quantified by the ImageJ software.

### Measurement of SOD activity and lipid peroxidation

We used the Total Superoxide Dismutase Assay Kit with WST-8 (Beyotime Biotechnology) to determine the SOD activity in kidney tissues. Briefly, 20 µL of tissue lysate supernatant, quantified using the BCA method, was reacted with 160 µL of WST-8/enzyme working solution and 20 µL of reaction starter working solution at 37 ^∘^C for 30 min. The absorbance was detected at 450 nm using a microplate reader (Biotek), and the total SOD (T-SOD) activity was expressed as units per milligram of protein (U/mgprot). Lipid peroxidation in kidney tissues was evaluated by measuring the malondialdehyde (MDA) content using a thiobarbituric acid assay kit (Nanjing Jiancheng Bioengineering Institute, China), and the optical density was detected at 532 nm using a microplate reader (Biotek). MDA content was expressed as nanomoles per milligram protein (nmoL/mgprot).

### Ethical statement

The clinical study was conducted in accordance with the Declaration of Helsinki and approved by the Ethics Committee of the Shuguang Hospital affiliated to SHUTCM (protocol code 2019-703-58-01). Written informed consent and clinical information of all participants were collected at the time of enrolment. The animal study protocol was approved by the Animal Experiment Ethics Committee of the SHUTCM (protocol code PZSHUTCM220725008).

### Statistical analysis

Analyses were performed with SPSS 26.0, and the results were visualized using GraphPad Prism 9.3. Normal distributions for continuous variables were determined using the Shapiro–Wilk test. All data are presented as mean ± SD or median with interquartile range and analyzed by one-way analysis of variance (ANOVA), Kruskal–Wallis *H* test, and Student’s *t*-test, as appropriate. The intraclass correlation coefficient (ICC) was used to analyze the reproducibility of the BOLD-MRI. The relationships between T2^*^ values and biochemical indicators were tested using the Pearson’s correlation coefficient. *P* < 0.05 was considered to be statistically significant.

## Results

### Reproducibility of BOLD-MRI in participants and rats

The high reproducibility (ICC > 0.89) of COT2^*^ and MET2^*^ in healthy individuals and patients with CKD has been demonstrated in our previous studies [12, 13]. In this study, COT2^*^ and MET2^*^ of all rats were analyzed by two radiologists, and the high reproducibility of both COT2^*^ (ICC ═ 0.917, *n* ═ 20) and MET2^*^ (ICC ═ 0.888, *n* ═ 20).

### Clinical characteristics of subjects

A total of 76 patients with CKD stages 1–5 and eight healthy subjects were included in the study. Participants were divided into three groups: HC (*n* ═ 8), CKD 1–3 (*n* ═ 49), and CKD 4–5 (*n* ═ 27). Their clinical characteristics are shown in (Table 1 and Figure 3C). There were significant differences among the three groups of COT2^*^ (*F* ═ 89.45, *P* < 0.0001), MET2^*^ (*F* ═ 36.73, *P* < 0.0001), and eGFR (*F* ═ 88.10, *P* < 0.0001). Moreover, significant differences in SCr, BUN, CysC, and Hemoglobin levels were also found among the three groups (all *P* < 0.0001). It is noteworthy that serum SOD was significantly different in the HC and CKD 1–3 groups compared to the CKD 4–5 group (HC vs CKD 4–5, *t* ═ 7.593, *P* < 0.0001; CKD 1–3 vs CKD 4–5, *t* ═ 5.618, *P* < 0.0001). However, there was no statistical difference in the serum SOD levels between the HC and CKD 1–3 groups (*t* ═ 1.774, *P* ═ 0.0815).

### Association of renal oxygenation, serum SOD, and eGFR in CKD

In previous studies, we found that renal oxygenation, evaluated by BOLD-MRI as COT2^*^ and MET2^*^, showed good correlations with eGFR in CKD [12, 13]. To further explore the relationships among hypoxia, oxidative stress, and renal function, Pearson correlations of COT2^*^, MET2^*^, eGFR, and serum SOD in patients with CKD are presented in (Figure 3D). The correlation coefficient was annotated as follows: 0.8–1.0, very strong correlation; 0.6–0.8, strong correlation; 0.4–0.6, moderate correlation; 0.2–0.4, weak correlation; and 0.0–0.2, very weak or no correlation. eGFR was significantly positively correlated with COT2^*^ (*r* ═ 0.8199, *P* < 0.0001), MET2^*^ (*r* ═ 0.7295, *P* < 0.0001), and serum SOD (*r* ═ 0.5541, *P* < 0.0001). Furthermore, there were strong positive correlations between serum SOD with COT2^*^ (*r* ═ 0.4911, *P* < 0.0001) and MET2^*^ (*r* ═ 0.4331, *P* < 0.0001) levels.

To avoid the effects of individualized clinical medication, we used 5/6 A/I rats to further investigate the relationship between renal hypoxia and oxidative stress in CKD. As shown in (Table 2 and Figure 4C), there were significant differences among the three groups of COT2^*^ (*F* ═ 19.74, *P* < 0.0001), MET2^*^ (*F* ═ 10.74, *P* ═ 0.0008), serum SOD (*F* ═ 16.64, *P* < 0.0001), and Ccr (*F* ═ 10.12, *P* ═ 0.0008). Ccr was positively associated with COT2^*^ (*r* ═ 0.7027, *P* ═ 0.0003), MET2^*^ (*r* ═ 0.7014, *P* ═ 0.0003), and serum SOD (*r* ═ 0.7771, *P* < 0.0001) levels. Additionally, serum SOD levels were positively associated with COT2^*^ (*r* ═ 0.7927, *P* < 0.0001) and MET2^*^ (*r* ═ 0.6792, *P* ═ 0.0005) levels (Figure 4D).

### Renal protection of losartan in CKD rats

**Figure 5.**
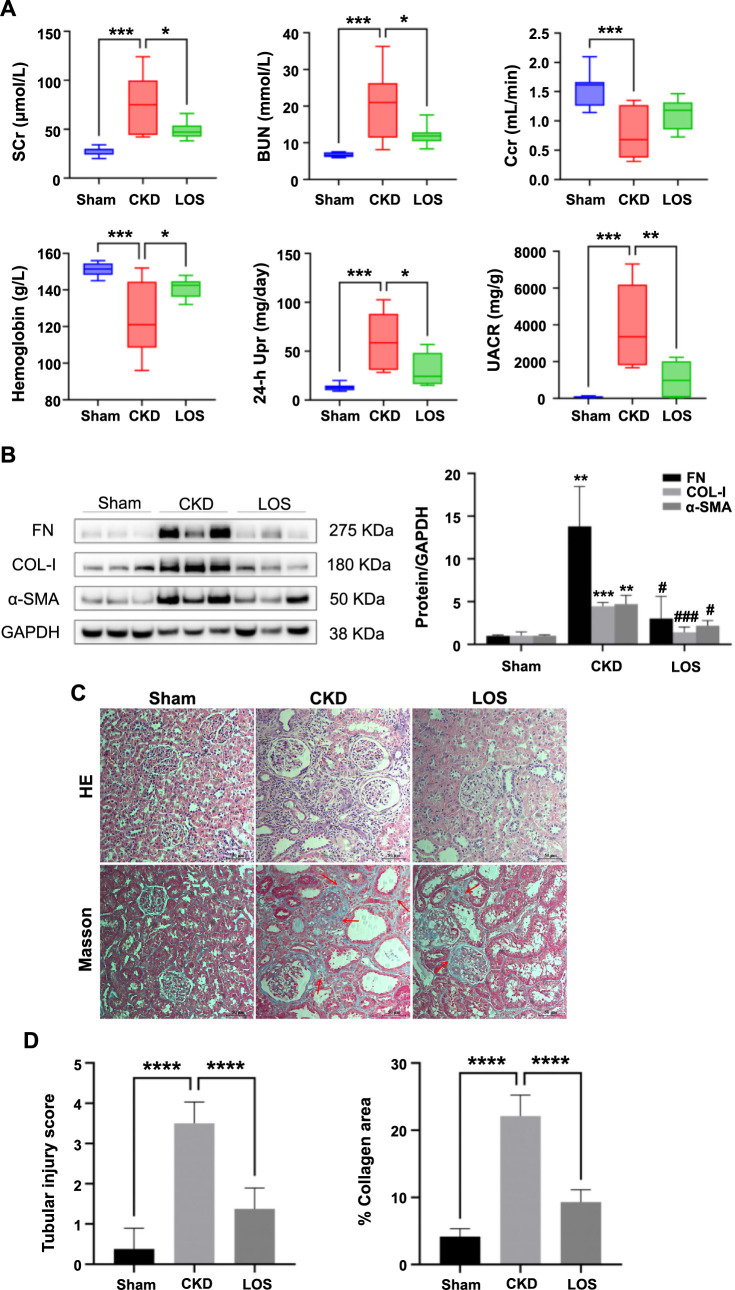
**The improvement of renal function and fibrosis by losartan in CKD rats.** (A) Comparisons of SCr, BUN, Ccr, 24-h Upr, UACR and hemoglobin among three groups (*n* ═ 8 per group); (B) The expression of FN, Col-I, and α-SMA proteins were determined by western blot (*n* ═ 3 per group); (C) Representative micrographs of HE and Masson’s trichrome staining (200× magnification), Representative fibrotic areas are marked with red arrows; (D) Assessment of tubular injury and collagen area (*n* ═ 8 per group). Data were analyzed by one-way ANOVA. Values are mean ± SD. ^*^P < 0.05; **P < 0.01; ***P < 0.001; ****P < 0.0001 (In addition, in Figure 5B, **P < 0.01; ***P < 0.001 vs Sham; ^#^*P* < 0.05, ^###^*P* < 0.001 vs CKD). SCr: Serum creatinine; BUN: Blood urea nitrogen; Ccr: Creatinine clearance rate; Upr: Urinary protein; UACR: Urinary albumin:creatinine ratio; LOS: Losartan; CKD: Chronic kidney disease; ANOVA: Analysis of variance.

In this study, we first assessed kidney function and proteinuria in rats from the three groups after an 8-week intervention to verify the renoprotective effect of losartan. As shown in (Figure 5A), SCr, BUN, 24-h Upr, and UACR in the CKD group were significantly higher than those in the Sham group (all *P* < 0.001), and Ccr and hemoglobin levels were significantly lower than those in the Sham group (*P* < 0.001). However, SCr, BUN, 24-h Upr, UACR, and hemoglobin levels of 5/6 A/I rats were significantly improved by losartan treatment (all *P* < 0.05).

Immunoblotting results showed that the expression of FN, Col-I, and α-SMA, markers for fibrosis, was significantly upregulated in the CKD group, but losartan treatment decreased the expression of FN, Col-I, and α-SMA in 5/6 A/I rats (Figure 5B). Additionally, HE and Masson staining analysis revealed more tubular injury and interstitial fibrosis in the CKD group, and losartan significantly attenuated the severity of renal fibrosis in 5/6 A/I rats (Figure 5C and 5D).

**Table 1:** Clinical characteristics of 84 subjects

	**HC (*n* ═ 8)**	**CKD 1–3 (*n* ═ 49)**	**CKD 4–5 (*n* ═ 27)**	***P* value**
Age (years)	40.13 ± 8.46	48.24 ± 10.85	60.19 ± 7.54	<0.0001
Gender (male/female)	5/3	24/25	13/14	0.7568
SCr (µmol/L)	60.88 ± 6.06	101.88 ± 31.20	276.63 ± 146.84	<0.0001
BUN (mmol/L)	4.06 ± 0.94	6.62 ± 1.98	14.55 ± 6.01	<0.0001
CysC (mg/L)	0.68 ± 0.02	1.22 ± 0.39	2.73 ± 0.61	<0.0001
eGFR (mL/min/1.73 m^2^)	112.75 ± 7.07	69.24 ± 24.90	20.64 ± 6.46	<0.0001
Hemoglobin (g/L)	142.46 ± 14.66	131.77 ± 20.02	116.90 ± 15.72	<0.0001
SOD (U/mL)	157.50 ± 9.86	143.73 ± 21.45	113.63 ± 23.95	<0.0001
COT2^*^ (ms)	70.15 ± 6.26	55.42 ± 6.42	40.24 ± 5.85	<0.0001
MET2^*^ (ms)	40.86 ± 3.94	30.99 ± 6.30	19.28 ± 3.78	<0.0001
24-h Upr (g/day)	—	0.69 (0.07–4.88)	2.16 (0.37–6.29)	<0.0001
*Cause of CKD (n)*				
Chronic glomerulonephritis	—	35	17	—
Hypertensive nephropathy	—	6	5	—
Other or unknown etiology	—	8	5	—

Data are presented as counts, mean (SD) or median (IQR). Data of gender and 24-h Upr were analyzed by Kruskal–Wallis *H* test and Student’s *t*-test, respectively, the others were analyzed by one-way ANOVA. HC: Healthy controls; SCr: Serum creatinine; BUN: Blood urea nitrogen; CysC: Serum cystatin C; SOD: Serum superoxide dismutase; eGFR: Estimated glomerular filtration rate; COT2^*^: Cortical T2^*^; MET2^*^: Medullary T2^*^; Upr: Urinary protein; UACR: Urinary albumin:creatinine ratio; CKD: Chronic kidney disease; ANOVA: Analysis of variance.

**Table 2:** Comparison of COT2^*^, MET2^*^, SOD, and Ccr from rats among the three groups

	**Sham (*n* ═ 8)**	**CKD (*n* ═ 8)**	**LOS (*n* ═ 8)**	**F**	***P* value**
COT2^*^ (ms)	53.64 ± 4.24	33.25 ± 5.60	43.60 ± 7.40	19.74	<0.0001
MET2^*^ (ms)	35.91 ± 2.16	23.26 ± 4.54	29.69 ± 7.05	10.74	0.0008
SOD (U/mL)	143.88 ± 11.43	102.38 ± 21.13	126.13 ± 6.96	16.64	<0.0001
Ccr (mL/min)	1.56 ± 0.30	0.79 ± 0.44	1.11 ± 0.27	10.12	0.0008

LOS: Losartan; COT2^*^: Cortical T2^*^; MET2^*^: Medullary T2^*^; SOD: Serum superoxide dismutase; Ccr: Creatinine clearance rate; CKD: Chronic kidney disease.

### Focus on renal hypoxia and oxidative stress in CKD treatment

**Figure 6.**
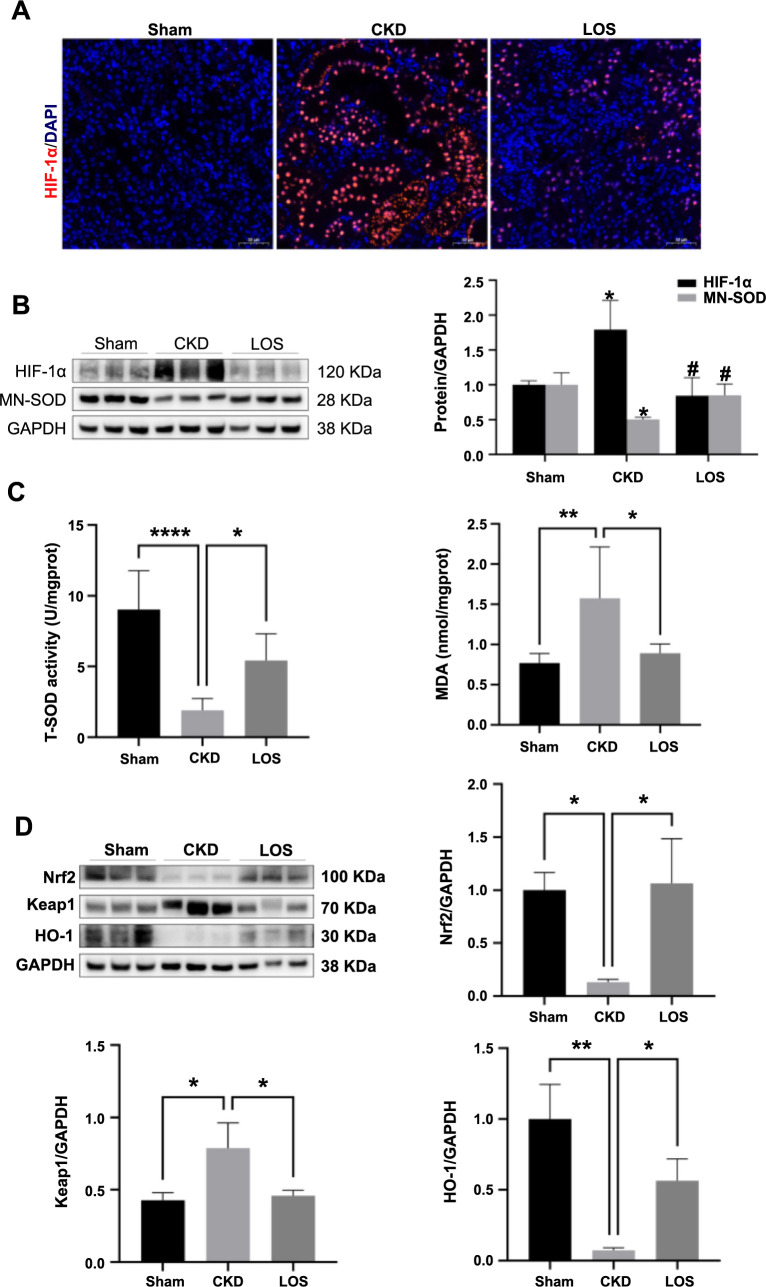
**The improvement of renal hypoxia and oxidative injury by losartan in CKD rats.** (A) Representative micrographs of immunofluorescence staining showing HIF-1α-positive area in kidney tissues among three groups (200× magnification); (B) Western blot and quantitative analyses for HIF-1α and MN-SOD in kidney tissues from three groups (*n* ═ 3 per group); (C) Relative expression levels of T-SOD activity and MDA content in three groups (*n* ═ 6 per group); (D) Western blot and quantitative analyses for Keap1, Nrf2, and HO-1 in kidney tissues from three groups (*n* ═ 3 per group). Data were analyzed by one-way ANOVA. Values are mean ± SD. ^*^P < 0.05; **P < 0.01; ****P < 0.0001 (In addition, in Figure 6B, ^*^P < 0.05 vs Sham; ^#^*P* < 0.05 vs CKD). LOS: Losartan; HIF-1α: Hypoxia-inducible factor-1α; SOD: Serum superoxide dismutase; MDA: Malondialdehyde; Keap1: Kelch-like ECH-associated protein 1; Nrf2: Nuclear factor erythroid 2-related factor 2; HO-1: Heme oxygenase-1; CKD: Chronic kidney disease; ANOVA: Analysis of variance.

To determine whether the renoprotective effect of losartan is consistent with the improvement in renal hypoxia and oxidative injury, we further evaluated the expression of hypoxia-related proteins and antioxidant capacity in the kidneys of the three groups. As shown in Figure 6A, the positive staining area of HIF-1α, a marker for hypoxia, in the CKD group was significantly larger than that in the Sham group, and losartan alleviated the expression of HIF-1α, which is consistent with the results of renal oxygenation evaluated by BOLD-MRI (Figure 4C). In addition, we performed immunoblotting to compare the expression of HIF-1α and MN-SOD in the three groups. Compared with the Sham group, the expression of HIF-1α protein was increased in the CKD group but was downregulated by losartan treatment. In contrast, the expression of MN-SOD protein decreased in the CKD group, and losartan treatment upregulated its expression (Figure 6B). In line with the above observations, T-SOD activity decreased and MDA content increased in 5/6 A/I kidneys (all *P* < 0.01), which was attenuated by losartan treatment (Figure 6C). These results indicate that the alleviation of renal injury in CKD is consistent with improvements in renal oxygenation and antioxidant capacity.

The nuclear factor erythroid 2-related factor 2 (Nrf2)-mediated antioxidant mechanism plays a critical role in defense against oxidative stress. We next explored whether losartan treatment enhances antioxidant capacity via the Keap1-Nrf2/HO-1 signaling pathway. Immunoblotting analysis showed that the expression of Nrf2 and HO-1 proteins was reduced in the CKD group, along with increased levels of Keap1, compared to those in the Sham group, which was attenuated by losartan treatment (Figure 6D). Therefore, the inhibition of oxidative stress by losartan in CKD rats may involve the activation of the Keap1-Nrf2/HO-1 signaling pathway.

## Discussion

This study was the first to thoroughly investigate the link between renal hypoxia, oxidative stress, and renal function in the progression and treatment of CKD in both clinical subjects and rat models. Accumulating evidence has revealed that chronic hypoxia and oxidative stress play important roles in the progression of CKD [3, 18]; however, the underlying relationship between them has not been well elucidated. In the present study, we quantified renal oxygenation in both patients with CKD and 5/6 A/I rats through BOLD-MRI and explored the relationship between hypoxia, oxidative stress, and renal function during the course of CKD. The results of the clinical study suggested that COT2^*^ and MET2^*^ gradually declined as CKD progressed, and eGFR had a significant positive correlation with COT2^*^ and MET2^*^. These findings agree with those of our previous studies showing that the severity of renal failure is associated with the degree of renal hypoxia [12, 13]. Furthermore, the serum SOD levels of the subjects also had significant positive correlations with eGFR, COT2^*^, and MET2^*^, suggesting that lower antioxidant capacity might be associated with renal failure and hypoxia.

Subsequently, we used 5/6 A/I rats, a typical intrarenal hypoxia model of CKD [15, 16], to study the role of renal hypoxia and oxidative stress in CKD progression. In line with the clinical findings, the levels of COT2^*^, MET2^*^, and serum SOD were decreased in CKD rats, and there were still positive correlations between serum SOD, COT2^*^ and MET2^*^ and Ccr, indicating that renal oxygenation and antioxidant capacity decreased with the deterioration of renal function. Additionally, we found that the levels of serum SOD, COT2^*^, and MET2^*^ increased with the improvement in renal function and fibrosis after losartan treatment. Next, we measured the expression of HIF-1α and SOD in renal tissues to focus on local hypoxia and oxidative stress in the treatment of CKD. HIF-1α is the main mediator of the hypoxic response, and increased HIF-1α levels can be used as a diagnostic marker for tissue hypoxia [19]. The results showed that the expression of HIF-1α protein was increased in 5/6 A/I kidneys but was reduced after losartan treatment. Oxidative stress is caused by a combination of increased ROS production and decreased antioxidant capacity [20]. As an antioxidant enzyme, a reduction in SOD activity has been linked to impaired antioxidant capacity. In this study, we found that T-SOD activity and MN-SOD protein expression were reduced in 5/6 A/I kidneys but were increased by losartan treatment. Furthermore, increased MDA content was detected in 5/6 A/I kidneys, which was also reduced by losartan treatment. MDA, a product of lipid peroxidation, is considered a biological marker of oxidative stress [21]. These findings demonstrate that renal hypoxia and oxidative stress were prevalent during the course of CKD, and losartan had some therapeutic effects on the improvement of renal oxygenation and antioxidant capacity.

Numerous antioxidant enzymes, such as SOD and HO-1, are regulated by Nrf2 and play a critical role in the defense against oxidative stress [22, 23]. Under oxidative stress, Nrf2 is released from bound Keap1 and translocates to the nucleus, activating the transcription of its target antioxidant genes to protect against oxidative damage [22]. Notably, our clinical data indicated that the level of serum SOD significantly declined from CKD stages 4–5, which was consistent with the downregulation of Nrf2, as reported in a study [24], suggesting that antioxidant capacity was reduced in severe kidney function impairment, especially in patients with CKD stages 4–5, and this could be reflected in decreased Nrf2. Aminzadeh et al. [25] reported increased oxidative stress with marked decreases in Nrf2 and its target proteins (HO-1 and MN-SOD) in 5/6 nephrectomy rats. Similarly, in the present study, decreased Nrf2 and HO-1 levels accompanied by an increase in Keap1 levels were also found in 5/6 A/I rat models of CKD, which is in line with the expression of these proteins in patients with severe renal injury as reported [26]. To some extent, this finding provides evidence for defining the severity of renal injury in a 5/6 A/I rat model of CKD.

Angiotensin II (Ang II), considered a crucial mediator of oxidative stress, stimulates ROS overproduction that aggravates inflammation, mitochondrial dysfunction, and renal fibrosis in the progression of CKD [27, 28]. Losartan, an Ang II antagonist, has been widely used and shown effective renoprotection in patients with CKD. In this study, we found that losartan restored Nrf2, HO-1, and SOD expression and downregulated Keap1 and HIF-1α expression in 5/6 A/I kidneys, indicating that the protective effects of losartan in renal hypoxia and oxidative stress may be associated with the activation of the Keap1-Nrf2/HO-1 signaling pathway. Mounting evidence suggests that Nrf2 activation improves GFR, vascular calcification, and renal fibrosis in CKD [29, 30]. Zhu et al. [28] also demonstrated the protective effect of Nrf2 activation on Ang II-induced mitochondrial injury in podocytes.

The present study has several limitations. First, the sample size of the clinical participants was relatively small, which could require more subjects in future studies to support the current findings. Second, we did not strictly limit drug administration in the enrolled patients, which could be more consistent with clinical reality. However, the administration of several drugs, such as diuretics and hypotensors, might affect renal oxygenation and oxidative stress. Third, the BOLD MR technique with breath-holding was used in this study to evaluate renal oxygenation, which could be difficult for children and elderly individuals to endure. We trained each participant carefully before MRI scanning, and subjects younger than 18 years or older than 75 years were excluded. Fourth, in order to keep a low-sodium diet before MRI scanning to prevent additional oxygen consumption from excessive sodium transport, the subjects’ diets were offered by the hospital cafeteria, and meal preparation followed the low-sodium dietary standards for CKD patients established by the Nutrition department during the study. However, their 24-h urinary sodium (UNa) data were not collected to be statistically analyzed. Finally, all rats were anesthetized by pentobarbital sodium before MRI scanning, and pentobarbital sodium might influence renal oxygenation [31]. Lastly, all rats were sedated with pentobarbital sodium prior to MRI scanning, which may have influenced renal oxygenation. However, the differences in dose among the rats were negligible and had minimal impact on the results of group comparisons.

## Conclusion

This study utilized BOLD-MRI to measure renal oxygenation in both CKD patients and rat models. Our findings confirm a correlation between renal hypoxia, oxidative stress, and renal function in CKD. Furthermore, the results demonstrate that losartan can partially improve reduced renal oxygenation and antioxidant capacity in CKD through the Keap1-Nrf2/HO-1 signaling pathway.

## Data Availability

The datasets generated and/or analyzed during the current study are not publicly available because of patient confidentiality, but are available from the corresponding author upon reasonable request.
